# Racial differences in the expression of inhibitors of apoptosis (IAP) proteins in extracellular vesicles (EV) from prostate cancer patients

**DOI:** 10.1371/journal.pone.0183122

**Published:** 2017-10-05

**Authors:** Salma Khan, Jennifer Simpson, James C. Lynch, David Turay, Saied Mirshahidi, Amber Gonda, Tino W. Sanchez, Carlos A. Casiano, Nathan R. Wall

**Affiliations:** 1 Center for Health Disparities & Molecular Medicine, Loma Linda University School of Medicine, Loma Linda, California; 2 Department of Basic Sciences, Loma Linda University School of Medicine, Loma Linda, California; 3 Cancer Center and Biospecimen Laboratory, Loma Linda University School of Medicine, Loma Linda, California; Southern Illinois University School of Medicine, UNITED STATES

## Abstract

African-American men with prostate cancer typically develop more aggressive tumors than men from other racial/ethnic groups, resulting in a disproportionately high mortality from this malignancy. This study evaluated differences in the expression of inhibitors of apoptosis proteins (IAPs), a known family of oncoproteins, in blood-derived exosomal vesicles (EV) between African-American and European-American men with prostate cancer. The ExoQuick^™^ method was used to isolate EV from both plasma and sera of African-American (n = 41) and European-American (n = 31) men with prostate cancer, as well as from controls with no cancer diagnosis (n = 10). EV preparations were quantified by acetylcholinesterase activity assays, and assessed for their IAP content by Western blotting and densitometric analysis. Circulating levels of the IAP Survivin were evaluated by ELISA. We detected a significant increase in the levels of circulating Survivin in prostate cancer patients compared to controls (P<0.01), with the highest levels in African-American patients (P<0.01). African-American patients with prostate cancer also contained significantly higher amounts of EVs in their plasma (P<0.01) and sera (P<0.05) than European-American patients. In addition, EVs from African-American patients with prostate cancer contained significantly higher amounts of the IAPs Survivin (P<0.05), XIAP (P<0.001), and cIAP-2 (P<0.01) than EVs from European-American patients. There was no significant correlation between expression of IAPs and clinicopathological parameters in the two patient groups. Increased expression of IAPs in EVs from African-American patients with prostate cancer may influence tumor aggressiveness and contribute to the mortality disparity observed in this patient population. EVs could serve as reservoirs of novel biomarkers and therapeutic targets that may have clinical utility in reducing prostate cancer health disparities.

## Introduction

African American (AA) men suffer from a disproportionately high incidence and mortality of prostate cancer (PCa) compared to European American (EA) men and men of other racial and ethnic backgrounds [1, 2]. While the basis for these health disparities is still not well understood, there is increasing awareness that they may result from the interplay between socioeconomic, lifestyle, and biological factors [3, 4]. In order to understand the molecular determinants contributing these disparities, it has become important to identify and characterize biomarkers of cancer development and biological factors that may contribute to the increased PCa mortality observed among AA men [4–6]. Accumulating evidence suggests that differential expression or activation of inflammatory, stress, and metabolic pathways that modulate immune responses to tumors or influence the balance between tumor cell proliferation and apoptosis could be a contributing factor to PCa progression in AA men [4–12].

Extracellular vesicles (EVs) have been described originating from numerous cellular populations including cancers and reside stably in biofluids of patients [13]. They are highly heterogeneous, variable in size, and contain what is believed to be a snapshot of the cellular contents of their cell of origin in their lumen and definitive molecules giving the EV distinct molecular and functional characteristics on their membrane surfaces [14]. Determining the difference between those produced from benign cells and those from pathological cells remains difficult. However, we and others have shown that the proteins residing both luminal and on the surfaces of EVs may facilitate uptake and function and in the case of cancer have been described to facilitate the aggressive phenotypes of cancer proliferation, invasion and therapeutic resistance to their recipient cells [14–17].

The inhibitor of apoptosis (IAP) protein Survivin has been implicated in apoptosis inhibition and regulation of mitosis in various cancer types, including PCa [18, 19]. Validating the cytoprotective mechanism of Survivin and other IAPs has become a priority because of the dramatic exploitation of this pathway by human tumors and its frequent association with unfavorable disease outcomes, and the recent identification of molecular antagonists of Survivin that are approaching clinical testing in cancer patients [20–22]. Our recent studies have shown that tumor-derived EVs contain Survivin and other IAPs, and play a key role in cell-to-cell communication within the tumor microenvironment [15, 16, 23, 24]. EVs are present in many biological fluids including saliva, cerebral spinal fluid (CSF), serum, plasma, and urine [25–29]. Our previous proteomic profiling studies suggested that serum-derived EV contents may be differentially expressed in a stage-dependent manner in PCa patients from different ethnicities [30]. As a result of these observations and our previous finding that Survivin, IAPs, and HSP70 are packaged in EVs and could be implicated as plausible biomarkers for PCa progression [15, 16, 23, 24], we hypothesized that Survivin and other stress survival proteins could be released differentially in AA and EA patients with PCa. In the present study we investigated the expression of four IAP proteins (Survivin, XIAP, cIAP-1, and cIAP2) in blood-derived EVs from AA and EA patients with PCa. Our results demonstrate a differential expression of exosomal IAP proteins between AA and EA patients with PCa.

## Materials and methods

### Patient plasma and serum samples

Plasma and serum samples were collected from AA (n = 41) and EA (n = 31) patients with PCa and from control patients without a diagnosis of cancer (n = 10). Samples were obtained under IRB-approved studies, following documentation of informed consent in accordance with policies at Loma Linda University Medical Center (LLUMC). The LLUMC Cancer Center Biospecimen Laboratory facilitated sample collection and processing. Samples were also acquired from Bioserve Biotechnologies (Beltsville, MD), a HIPPA-compliant biorepository that operates through IRB-approved collection protocols. Samples from both sites were provided with a limited data set of protected health information. Available clinicopathological characteristics of the patient samples used in this study are listed in S1 Table.

### Quantification of Survivin in patient plasma and serum by ELISA

Plasma and serum samples were assayed for the presence of Survivin using the Quantikine Human Survivin Immunoassay kit (R&D Systems, Inc., Minneapolis, MN) as we described previously [15] and according to the manufacturer’s instructions.

### Extracellular vesicle isolation

EVs were isolated from patient plasma or serum using ExoQuick^™^ (System Biosciences, Mountain View, CA, USA) according to the manufacturer’s instructions. ExoQuick^™^ is a polymer that is increasingly being used as an effective method for isolating EVs from cell lines and clinical fluids [15, 26, 31]. Briefly, plasma or sera collected from patients were centrifuged at 3,000 x g for 15 minutes to remove any cells or cell debris. Collected supernatants were then filtered through a 0.45 μm filter to further eliminate cellular debris. Filtered plasma/sera was mixed with 1:1 ratio of Exoquick^™^ solution, incubated at 4°C for 2 hours, and centrifuged at 1500 x g for 5 minutes. The supernatants were then aspirated without disturbing the pellet. Pellets containing the EV fractions were reconstituted with 500 μl of double distilled water.

### Extracellular vesicle quantification

To quantify the amount of EV released, we assessed the activity of acetylcholinesterase, an enzyme that is specific to these vesicles. Acetylcholinesterase activity correlates with EV amount and was assessed as described by Savina et al. [32]. Briefly, 40 μl of the EV fractions were mixed with 110 μl of PBS. Next, 37.5 μl of this PBS-diluted EV fraction was added to individual wells of a 96-well flat-bottomed microplate and 1.25 mM acetylthiocholine, and 0.1 mM 5,5'-dithiobis(2-nitrobenzoic acid) were then added to EV fractions to a final volume of 300 μl. Change in absorbance at 412 nm was monitored every 5 minutes for 30 minutes.

### Western blot analysis

EV preparations were solubilized using lysis buffer (50 mM Tris (pH 7.5), 1% NP40, 0.25% deoxycholic acid, 150 nM sodium chloride, 1 mM PMSF, 10 μg/ml aprotinin/leupeptin/pepstatin, 20 mM sodium fluoride, 0.02 mM EGTA, and 1 mM EDTA (pH 8.0)). The BCA assay (Pierce, Rockford, IL) was used to determine protein concentrations. Exosomal proteins (20–40 μg) were separated by electrophoresis using 4–12% or 4–15% gradient Tris polyacrylamide gels (Life Technologies, Grand Island, NY) and transferred onto polyvinyl difluoride (PVDF) membranes (Millipore, Billerica, MA). After blocking with 5% nonfat dry milk in 1XPBST (phosphate buffered saline with 0.1% Twin 20), membranes were probed using the following antibodies: rabbit polyclonal anti-Survivin (Novus, Littleton, CO, cat# NB100-56167), XIAP, cIAP-1, cIAP-2 (Cell Signaling, Danvers, MA, cat #2042, #4952S, #3130S, respectively), Lamp 1 (Abcam, Cambridge, MA, cat #ab25245) and goat anti-mouse and anti-rabbit immunoglobulin (LICOR, Lincoln, Nebraska, cat #s 926–32211 and 925–32210). Immunoreactive bands were detected using the Odyssey imaging system (LICOR) and quantified using ImageQuant software.

### Statistical analysis

Multiple comparisons among different groups were calculated by using Multiple Analysis of Variance (MANOVA). Student’s t-test (two tailed) was used to evaluate the significance of changes between control groups and experimental groups. Probability values p<0.05 were considered statistically significant.

## Results

### Differential Survivin levels in sera/plasma from AA and EA patients with prostate cancer

**Fig 1:**
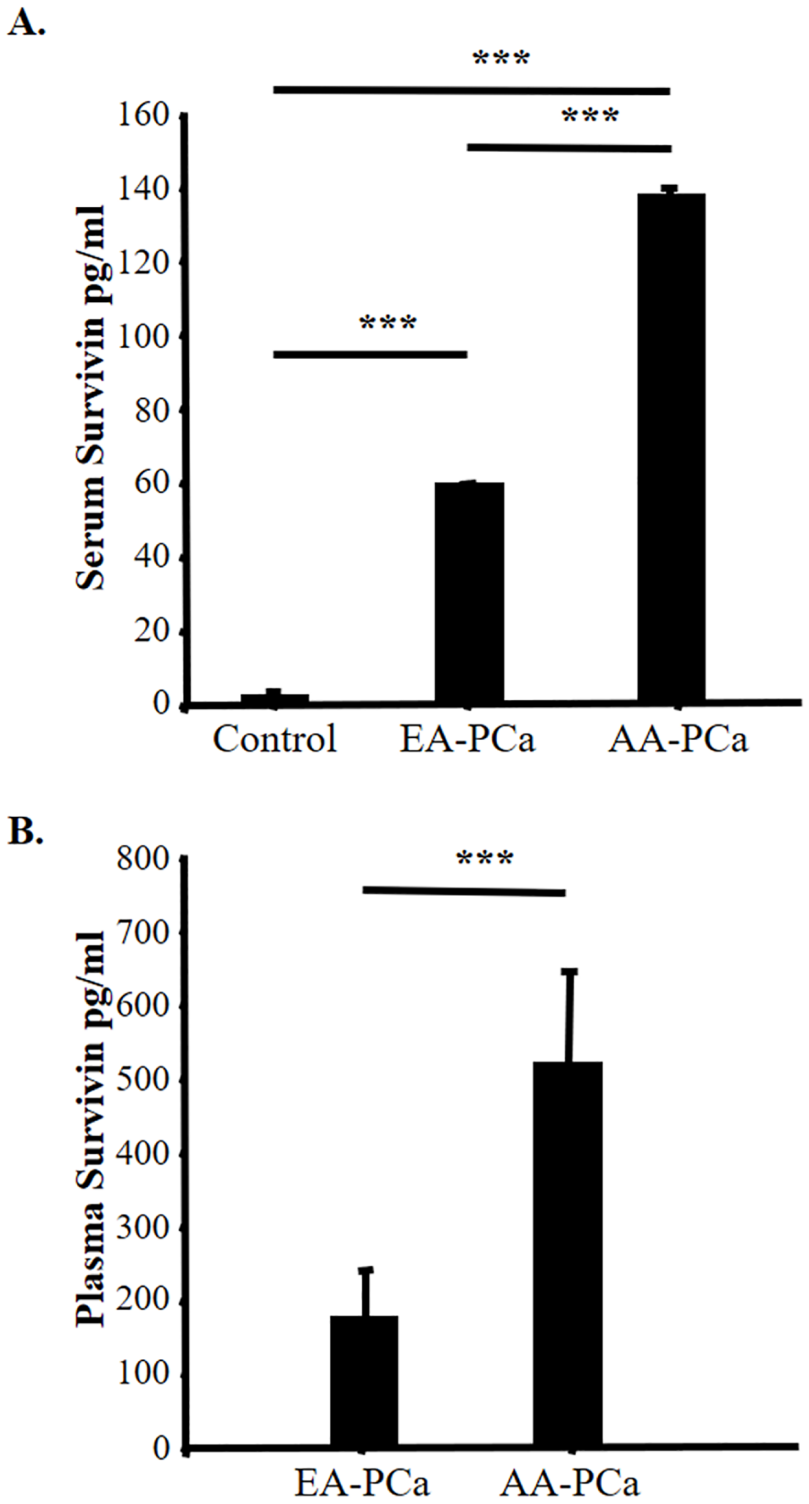
A. Survivin levels were detected in sera derived from European American (EA) (n = 17) and African American (AA) (n = 21) patients with prostate cancer (PCa), as well as from control sera (n = 10) from individuals with no diagnosis of cancer. Comparisons between groups were analyzed by MANOVA (** p < 0.01). B. Survivin levels were detected in plasma derived from EA (n = 10) and AA (n = 12) patients with PCa. Comparisons were analyzed by Student’s t-test (*** p < 0.001). (*N6 and *N14 are Hispanics; *N12 is Asian, we have excluded those from the analysis).

In a previous study we observed a significant elevation in the levels of Survivin circulating in plasma and sera from PCa patients, compared to healthy controls [15]. To determine if there is a race-associated differential release of Survivin, plasma and serum samples were collected from EA and AA PCa patients and used to quantify the levels of this protein by ELISA. Control subjects without a diagnosis of cancer were also analyzed. Survivin levels were significantly higher (P<0.01) in the PCa-derived serum from both patient groups than in those from the control group (Fig 1A). In addition, Survivin levels were higher in plasma than in sera for both EA-PCa and AA-PCa patients (Fig 1B). Interestingly, Survivin levels were significantly higher (P<0.01) in AA-PCa patients than EA-PCa patients (Fig 1A and 1B).

### Differential EV release in AA and EA patients with prostate cancer

**Fig 2:**
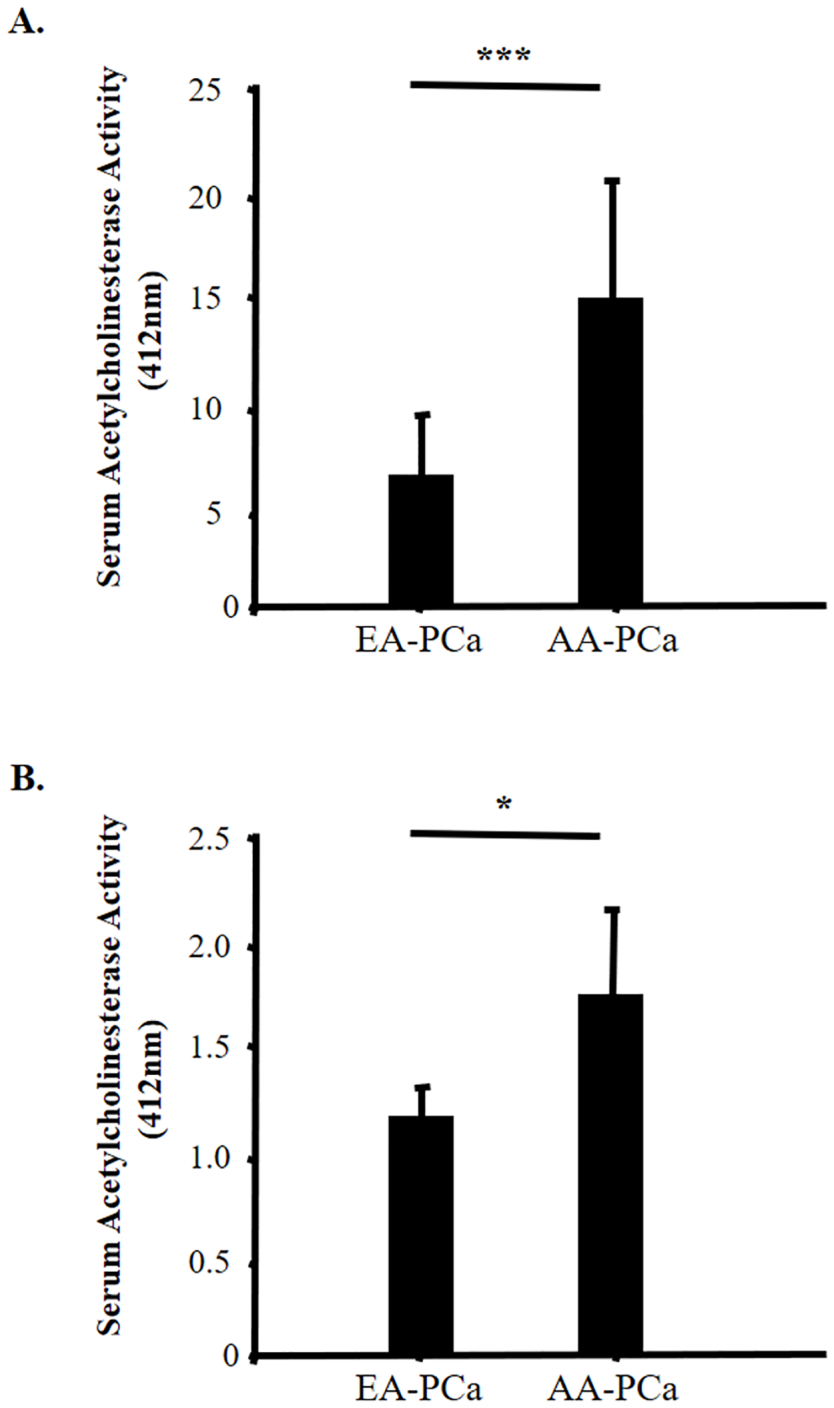
Acetylcholinesterase (AChE) activity assay was used to indirectly detect EV quantities from plasma (A) and serum (B)-derived EV. **A**. EVs were isolated from plasma of European American (EA) (n = 8) and African American (AA) (n = 17) patients with prostate cancer (PCa). **B**. EVs were isolated from sera of EA (n = 23) and AA (n = 24) patients with PCa. Comparative analysis was established using Student’s t-test (**p < 0.01 and **p < 0.05). (*N6 and *N14 are Hispanics; *N12 is Asian, we have excluded those from the analysis).

The release of Survivin-containing EVs has been previously detected by our group, and others, in both plasma and serum samples from PCa patients [15, 18, 30]. To determine if the differentially elevated levels of circulating Survivin in AA-PCa patients was reflective of differentially released EVs in these two groups, we collected EVs from both serum and plasma, derived from AA-PCa and EA-PCa patients. The amount of released EV was indirectly and semi-quantitatively assessed using the acetylcholinesterase (AChE) activity assay [32]. Consistent with our previous observations [15], we isolated significantly larger (P<0.05) quantities of EV from plasma samples than from serum samples, as suggested by the increased AChE activity in plasma compared to serum (Fig 2A and 2B). Interestingly, in line with our observation of higher Survivin levels in sera and plasma from AA-PCa patients compared to EA-PCa patients (Fig 1), there was also a significantly elevated (**3–4 fold**) AChE activity, indicative of increased EV release, in AA-PCa derived plasma samples compared to EA-PCa derived plasma (P<0.01) (Fig 2A). A similar trend was observed when serum-derived EVs were compared, as sera from AA-PCa patients had increased AChE activity (**2–3 fold**) compared to EA-PCa sera (P<0.05) (Fig 2B).

### Plasma and sera-derived EV contain Survivin and other IAPs

**Fig 3:**
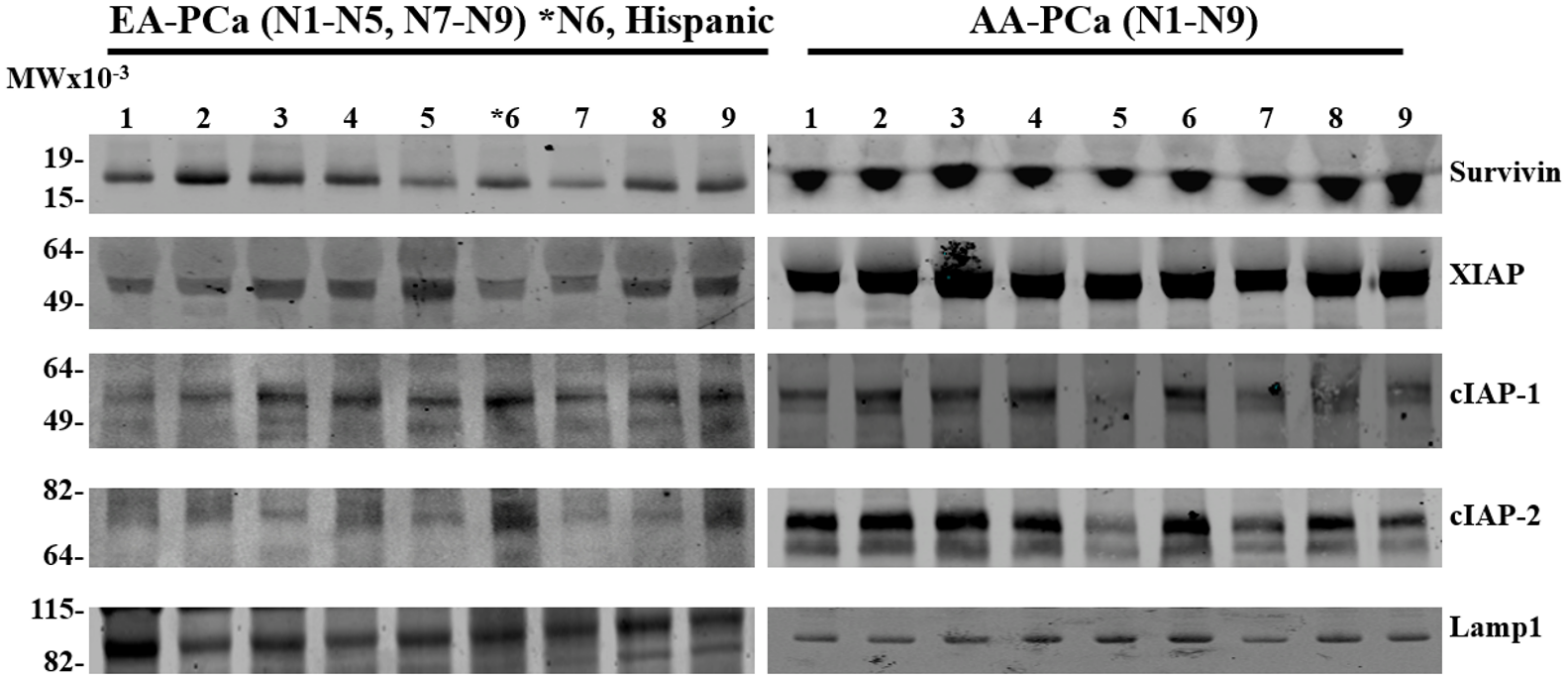
Representative Western blots showing the IAP levels in EV derived from individual European American (EA) (N1-N5) & (N7-N9), *N6 and African American (AA) (N1-N9) patients with prostate cancer (PCa). Specific antibodies against Survivin, XIAP, cIAP-1, cIAP-2, and Lamp1 were used for the Western blotting analysis of total exosomal proteins. The blots from both patient groups were processed under identical conditions; Lamp 1 was used as loading control. (*N6, Hispanic.) (Both blots were done side by side in the same gel running and transferring apparatus, blocking, washing buffers, and antibody incubations were done in the same time, in the same incubating trays under the identical exposure to keep the consistencies.)

**Fig 4:**
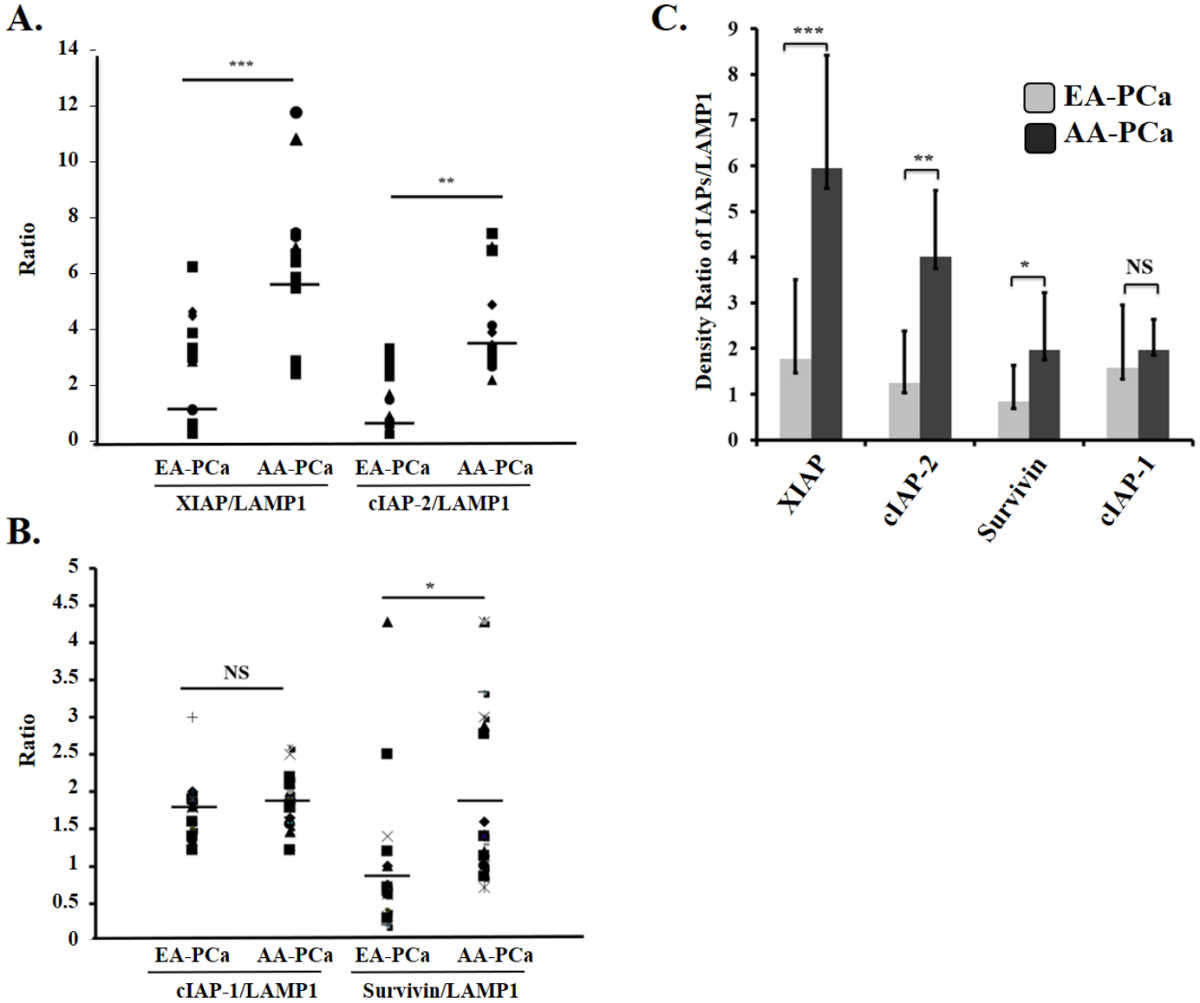
Densitometric analysis of Western blots showing the EV expression of XIAP, cIAP-2, Survivin, and cIAP-1 in European American (EA) and African American (AA) patients with prostate cancer (PCa). **A**. Scatter plot analysis of protein band density ratios of XIAP/LAMP-1 and cIAP-2/Lamp-1 derived from individual serum/plasma samples of EA (n = 31) and AA (n = 41) PCa patients (-, mean values). **B**. Scatter plot analysis of protein band density ratios of cIAP-1/LAMP-1 and Survivin/Lamp-1 derived from individual serum/plasma samples of EA (n = 31) and AA (n = 41) PCa patients (-, mean values). **C**. Proportion analysis of all IAPs/Lamp1 ratios in EV samples from EA (n = 31) and AA (n = 41) PCa patients (XIAP, (***p<0.001; cIAP-2, **p<0.01; Survivin, *p<0.05; cIAP-1, NS, not significant). (*N6 and *N14 are Hispanics; *N12 is Asian, we have excluded those from the analysis).

Given the differentially increased levels of Survivin and EVs in AA-PCa patients compared to EA-PCa patients, we sought to assess by Western blotting the amount of IAPs in EV from patient plasma and sera. As loading control in these experiments we used lysosomal-associated membrane protein 1 (LAMP1), a known vesicular protein that is commonly used as control in western blotting analysis of EV contents [15, 26]. EVs isolated from the sera or plasma of AA-PCa patients exhibited increased expression of Survivin, XIAP, and cIAP-2 compared to EVs collected from EA-PCa patients (Fig 3 and S1, S2 and S3 Figs). Densitometric analysis of blots corresponding to the total number of patient samples showed differences in the ratios of XIAP/Lamp1, cIAP-2/Lamp1, and Survivin/Lamp1 between the two patient groups, with AA-PCa patients showing significantly higher expression of XIAP (P<0.001), c-IAP-2 (P<0.01), and Survivin (P<0.05) (Fig 4A–4C). The expression levels of XIAP and c-IAP-2 in the EVs also appeared to be higher than those of Survivin in the AA-PCa group (Figs 3 and 4, S1, S2 and S3 Figs). However, there was no significant difference in exosomal cIAP-1 expression between the two patient groups.

### Clinicopathological considerations

Limited clinicopathological data were acquired for the AA- and EA-PCa patients included in this study (S1 and S2 Tables). Rates of recurrence were correlated with Survivin, XIAP, cIAP-1 and cIAP-2 expression. We found that the degree of Survivin expression was related to recurrence rate but there was no correlation found with other IAPs (S2 Table). Our statistical analysis did not reveal a significant difference between the two groups in terms of their Gleason scores (AA: 23% Gleason 6, 52% Gleason 7, 23% Gleason 8; EA: 23% Gleason 6, 53% Gleason 7, 11% Gleason 8 and 9), age, or PSA levels. In comparing the Gleason scores with individual IAPs, XIAP showed a steady increase as the Gleason score increased which was of significance (p<0.05) in AA patients but not in EA-PCa patients (S1 Table and S4 Fig). After careful ethnicity search, EA N6 and EA N14 were found to be Hispanics and EA N12 Asian, thus we removed them from the analysis.

## Conclusions

Recent advances in the study of biological determinants contributing to PCa health disparities have revealed significant differences in tumor biology between AA- and EA-PCa patients [4–6]. These include anatomical differences in tumor localization within the prostate as well as differences in tumor gene expression patterns, DNA methylation patterns, chromosomal alterations, microRNA profiles, genetic polymorphisms, mitochondrial DNA content, oncoprotein tissue expression, and advanced glycation end products [4–12]. In this study, we report for the first time significantly higher circulating levels of EVs as well as exosomal IAPs in serum/plasma from AA-PCa patients compared to EA-PCa patients and controls with no PCa diagnosis. This observation is in agreement with previous observations linking the tissue expression and extracellular circulation of IAP proteins with PCa progression and drug resistance [15, 33]. Consistent with documented Survivin expression being associated with unfavorable clinicopathological parameters [18, 34], extracellular trafficking of Survivin and other IAPs throughout the tumor microenvironment could augment tumor aggressive properties while prohibiting or minimizing therapeutic results.

PCa has been shown to be more aggressive and hence more challenging to treat in AA compared to EA patients [35–37]. Therefore, in addition to more specific diagnostic markers, prognostic and therapeutic markers are also needed to act as surrogate endpoints in forecasting disease severity, choosing treatments, and monitoring response to therapies [38]. In a previous proteomics study we reported that EV-derived proteins purified from PCa patients serum were differentially expressed in ethnically diverse populations, with sets of proteins that were unique to either AA or EA patients, as well as proteins that were common to both groups [30]. Consistent with these results, the present study shows that while both AA- and EA-PCa patients have increased circulating levels of EV containing IAPs, compared to controls with no PCa diagnosis, the EVs from AA patients showed the highest IAP levels. Previously, we showed that higher levels of EV Survivin in the serum from PCa patients correlated with increased resistance to docetaxel therapy [15]. It is plausible that increased exosomal release of XIAP, Survivin and cIAP-2, and perhaps other survival and stress proteins might be linked to increased tumor aggressiveness and chemoresistance in AA-PCa patients.

A limitation of this study was the relatively small sample size, which prevented us from establishing significant correlations in both groups between clinicopathological parameters (e.g. Gleason scores, PSA, clinical stage, etc) and increased expression of these IAPs in EV. Further studies with large cohorts of AA- and EA-PCa patients that have progressed to metastatic castration resistant PCa and taxane resistance are guaranteed to investigate a link between increased exosomal IAP expression and PCa progression and chemoresistance in AA-PCa patients.

Validating a cytoprotective mechanism driven by Survivin and other IAPs has become a research priority because of the dramatic exploitation of this pathway in human tumors [18, 19]. Survivin’s frequent association with unfavorable disease outcomes [39, 40] has led to the recent identification of molecular antagonists of this protein that are approaching clinical testing in cancer patients [19–22]. Deregulation of apoptosis is thought to invariably occur in human cancer, and facilitate the acquisition of deleterious cancer traits, including increased cell proliferation and clonogenicity, loss of tumor suppressor genes, angiogenic changes, and immortalization [41, 42]. In addition, suppression of both apoptotic and non-apoptotic cell death pathways by pro-survival and stress proteins have been linked to chemotherapy resistance in PCa [43, 44]. It would be of interest to evaluate whether cell survival pathways associated with chemoresistance are differentially upregulated in prostate tumors from AA men compared to those of EA men.

In summary, this study augments our ongoing work on EV-contained Survivin [15, 16, 23] by showing that other members of the IAP family, in this case cIAP2 and XIAP, may also be involved in differentially modulating the prostate tumor microenvironment in a race-related context, potentially contributing to racial disparities in PCa mortality. Further studies with larger patient cohorts with comprehensive clinical information are necessary to evaluate the correlation between exosomal IAPs with clinicopathological parameters of PCa in AA and EA patients. In future studies, we will further evaluate the differential release and activation of these and other EV-derived pro-survival and stress proteins in larger, diverse cohorts of PCa patients, and establish if they correlate with increased resistance to therapy in a race-related manner. EV may prove to be very important tumor survival protein reservoirs influencing PCa responses to therapy and the racial disparities in mortality associated with this malignancy. Their study could potentially lead to the development of novel tools to predict PCa progression and the differentially aggressive prostate tumor behavior observed between AA and EA men.

## Supporting information

S1 Fig IAP levels in EA and AA PCa patients. Western blots showing the IAP levels in EV derived from individual European American (EA) (N10-N11, N13) *N12 and African American (AA) (N10-N14) patients with prostate cancer (PCa). Specific antibodies against Survivin, XIAP, cIAP-1, cIAP-2, and Lamp1 were used for the Western blotting analysis of total EV proteins. The blots from both patient groups were processed under identical exposure conditions. (*N12, Asian). (Both blots were done side by side in the same gel running and transferring apparatus, blocking, washing buffers, and antibody incubations were done in the same time, in the same incubating trays under the identical exposure to keep the consistencies.
(TIF)

S2 Fig Western blots showing the IAP levels in EV derived from individual European American (EA) (N15-N31), (*N14) and African American (AA) (N15-N32) patients with prostate cancer (PCa). Specific antibodies against Survivin, XIAP, cIAP-1, cIAP-2, and Lamp1 were used for the Western blotting analysis of total EV proteins. The blots from both patient groups were processed under identical exposure conditions. (*N14, Hispanic) (Both blots were done side by side in the same gel running and transferring apparatus, blocking, washing buffers, and antibody incubations were done in the same time, in the same incubating trays under the identical exposure to keep the consistencies).
(TIF)

S3 Fig Differential IAP levels in African American PCa patients. Western blots showing the IAP levels in EV derived from the remaining African American (AA) (N33-N41) patients with prostate cancer (PCa). Specific antibodies against Survivin, XIAP, cIAP-1, cIAP-2, and Lamp1 were used for the Western blotting analysis of total exosomal proteins. The blots from both patient groups were processed under identical conditions. (All these blots were done side by side in the same gel running and transferring apparatus, blocking, washing buffers, and antibody incubations were done in the same time, in the same incubating trays under the identical exposure to keep the consistencies.).
(TIF)

S4 Fig IAPs and Gleason scores. Analysis of IAPS (Survivin, XIAP, cIAP-1, and cIAP-2) density and gleason score. All PCa patients from Gleason 6–9 (%) were correlated by western blot density analysis. Significance defined by a p value < 0.05.
(TIF)

S1 Table Demographics of patients in both EA-PCa and AA-PCa groups. (DOCX)

S2 Table Rate of recurrence in both ethnicities and IAP expression in EV. (DOCX)
