# Supplementary figures and images for: Racial differences in the expression of inhibitors of apoptosis (IAP) proteins in extracellular vesicles (EV) from prostate cancer patients
Source: PLoS One. 2017 Oct 5;12(10):e0183122. doi: 10.1371/journal.pone.0183122 (PMC5628787; doi:10.1371/journal.pone.0183122)

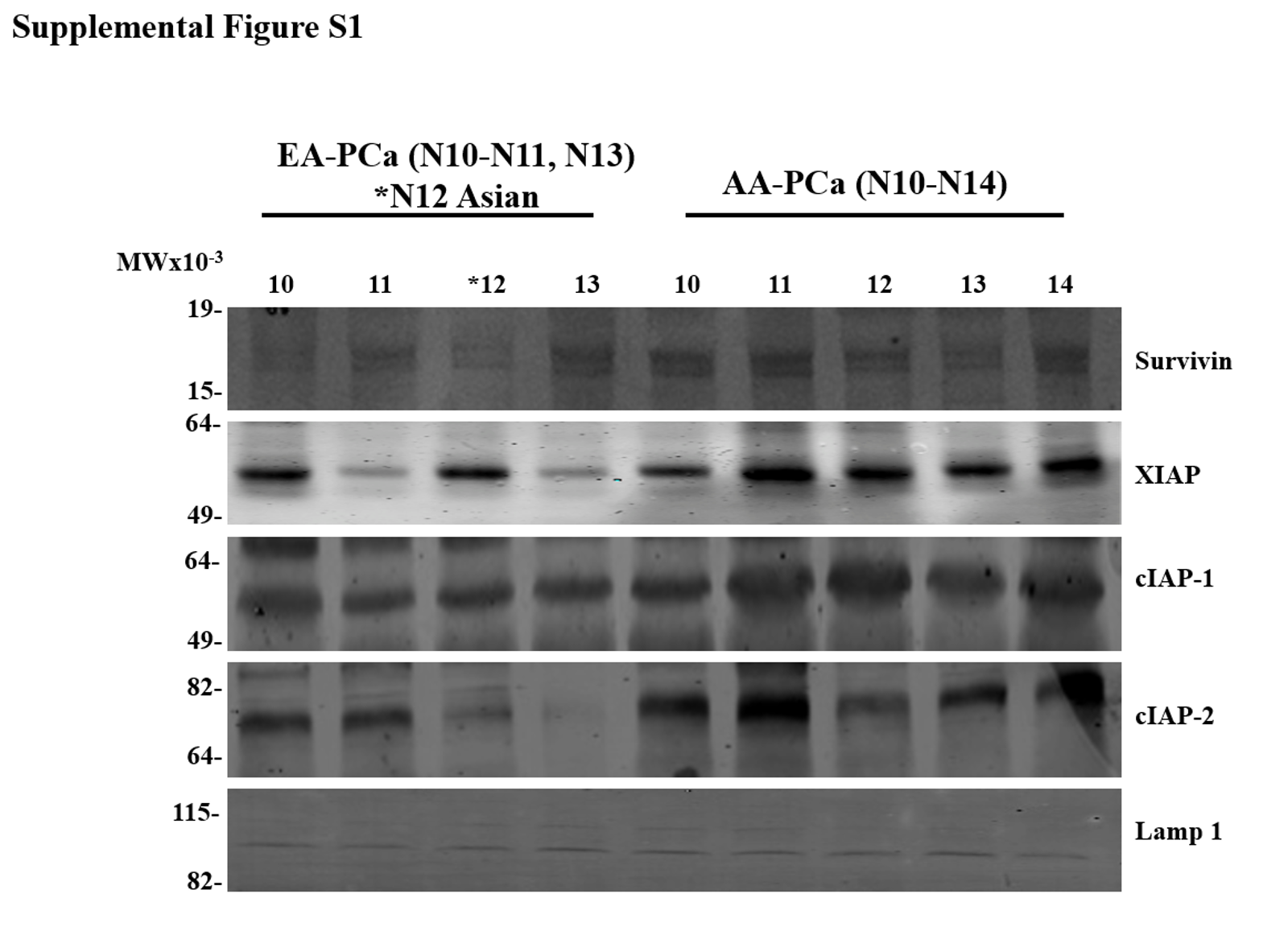

Supplement: S1 Fig — Western blots showing the IAP levels in EV derived from individual European American (EA) (N10-N11, N13) *N12 and African American (AA) (N10-N14) patients with prostate cancer (PCa). Specific antibodies against Survivin, XIAP, cIAP-1, cIAP-2, and Lamp1 were used for the Western blotting analysis of total EV proteins. The blots from both patient groups were processed under identical exposure conditions. (*N12, Asian). (Both blots were done side by side in the same gel running and transferring apparatus, blocking, washing buffers, and antibody incubations were done in the same time, in the same incubating trays under the identical exposure to keep the consistencies. (TIF) [file pone.0183122.s001.tif]

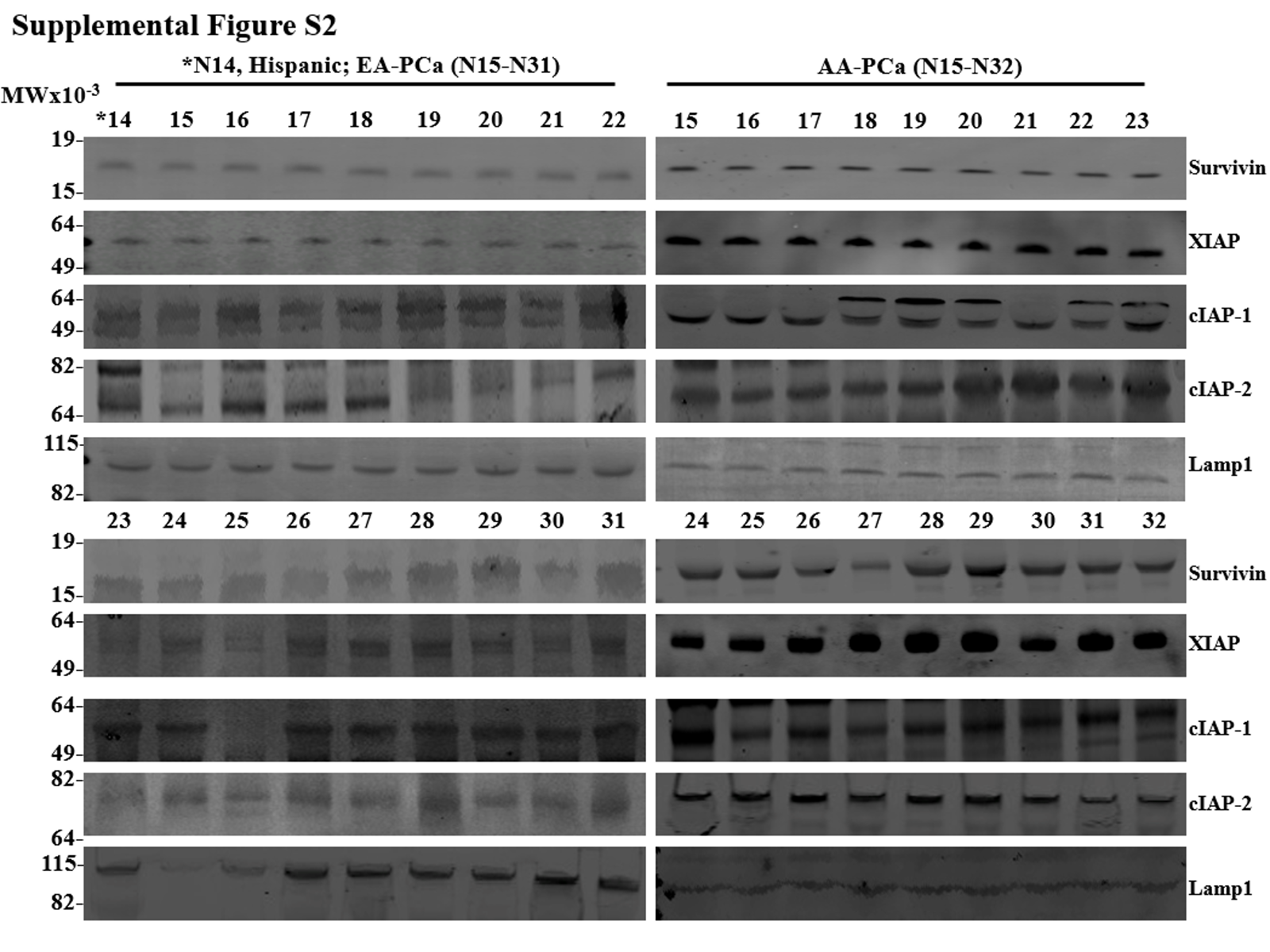

Supplement: S2 Fig — Specific antibodies against Survivin, XIAP, cIAP-1, cIAP-2, and Lamp1 were used for the Western blotting analysis of total EV proteins. The blots from both patient groups were processed under identical exposure conditions. (*N14, Hispanic) (Both blots were done side by side in the same gel running and transferring apparatus, blocking, washing buffers, and antibody incubations were done in the same time, in the same incubating trays under the identical exposure to keep the consistencies). (TIF) [file pone.0183122.s002.tif]

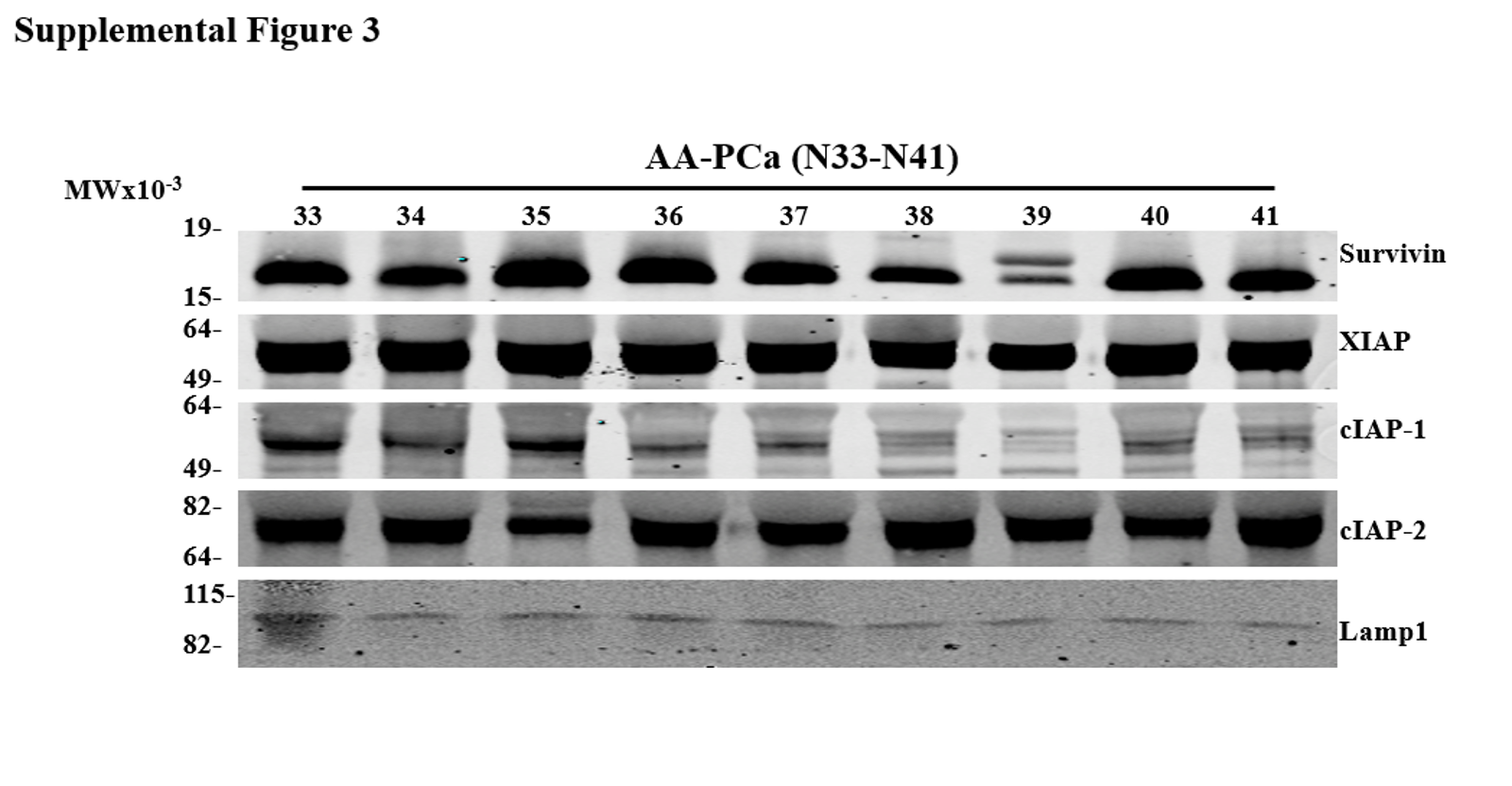

Supplement: S3 Fig — Western blots showing the IAP levels in EV derived from the remaining African American (AA) (N33-N41) patients with prostate cancer (PCa). Specific antibodies against Survivin, XIAP, cIAP-1, cIAP-2, and Lamp1 were used for the Western blotting analysis of total exosomal proteins. The blots from both patient groups were processed under identical conditions. (All these blots were done side by side in the same gel running and transferring apparatus, blocking, washing buffers, and antibody incubations were done in the same time, in the same incubating trays under the identical exposure to keep the consistencies.). (TIF) [file pone.0183122.s003.tif]

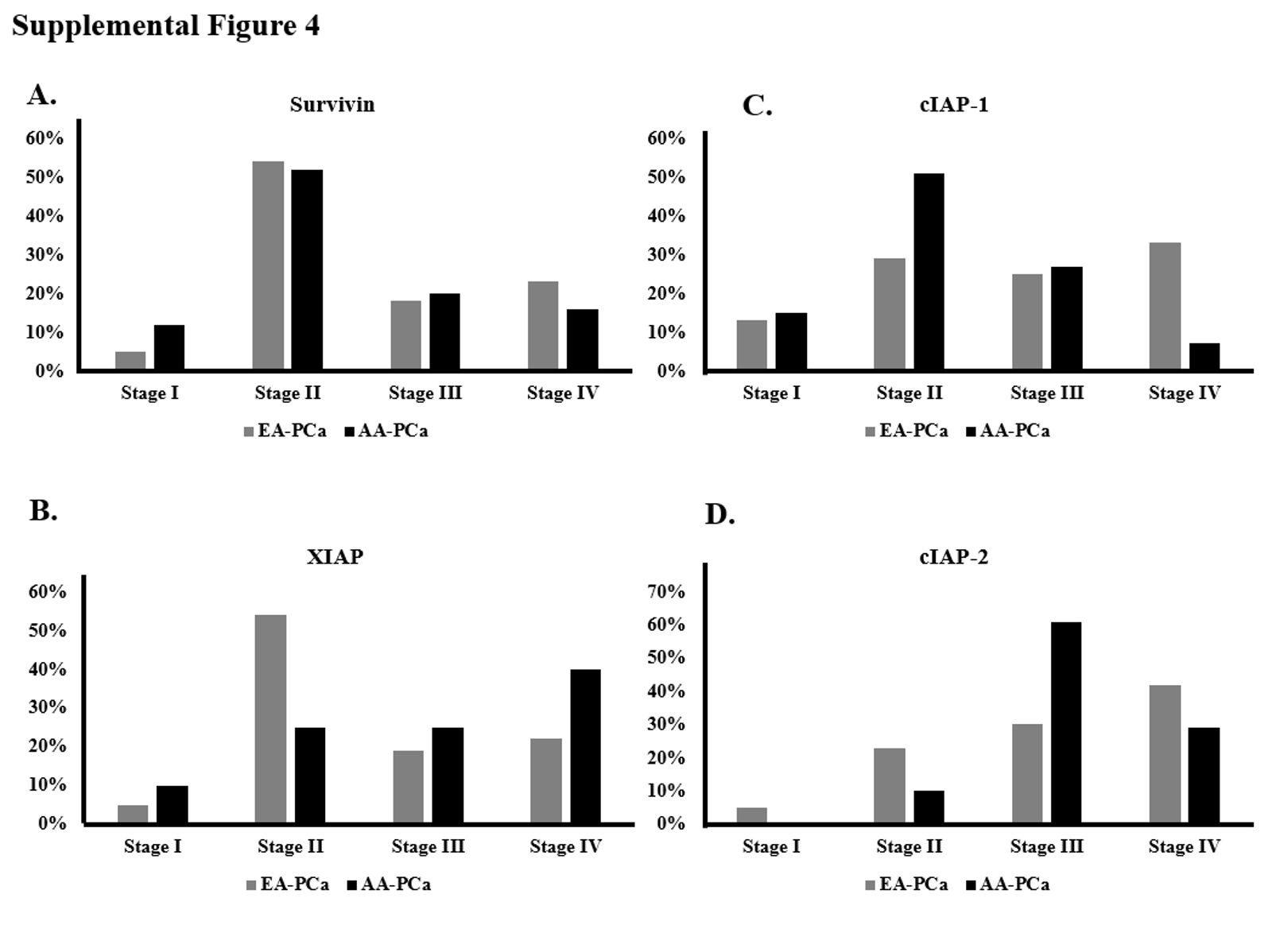

Supplement: S4 Fig — Analysis of IAPS (Survivin, XIAP, cIAP-1, and cIAP-2) density and gleason score. All PCa patients from Gleason 6–9 (%) were correlated by western blot density analysis. Significance defined by a p value < 0.05. (TIF) [file pone.0183122.s004.tif]
