# Supplementary material for: Racial differences in the expression of inhibitors of apoptosis (IAP) proteins in extracellular vesicles (EV) from prostate cancer patients
Source: PLoS One. 2017 Oct 5;12(10):e0183122. doi: 10.1371/journal.pone.0183122 (PMC5628787; doi:10.1371/journal.pone.0183122)
Supplement: S1 Table — (DOCX) [file pone.0183122.s005.docx]

**S1 Table. Demographics of patients in both EA-PCa and AA-PCa groups.**

| **Patient** | **Age** | **Race / Ethnicity** | **PSA** | **Gleason** | **GSC Total** | **Stage** | **Family history** | | **Prostat-ectomy** | | | **Biochem. Recurrence** | | | **Treatment** | | |  |
| --- | --- | --- | --- | --- | --- | --- | --- | --- | --- | --- | --- | --- | --- | --- | --- | --- | --- | --- |
| PCa-EA1 | 48 | White | 2.3 | -- | -- | II | -- | Yes | | No | | | ?? | | |  |  |  |
| PCa-EA2 | 63 | White | 6.2 | 3+3 | 6 | II | None | Yes | | -- | | |  | | |  |  |  |
| PCa-EA3 | 64 | White | 3.55 | 3+3 | 6 | -- | None | Yes | | No | | |  | | |  |  |  |
| PCa-EA4 | 57 | White | 6.7 | 4+3 | 7 | -- | None | ? | | ? | | |  | | |  |  |  |
| PCa-EA5 | 73 | White | N/A | 4+4 | 8 | II | F1 | Yes | | No; died | | |  | | |  |  |  |
| PCa-EA6 | 64 | Hispanic | 13.5 | 3+4 | 7 | II | F2 | Yes | | No | | |  | | |  |  |  |
| PCa-EA7 | 63 | White | 5.9 | 3+4 | 7 | -- | -- | Yes | | No | | |  | | |  |  |  |
| PCa-EA8 | 62 | White | 16 | 4+5 | 9 | II | Fo2 | Yes | | -- | | |  | | |  |  |  |
| PCa-EA9 | 65 | White | 14.7 | 4+4 | 8 | III | F2 | Yes | | No | | | Radiation? | | |  |  |  |
| PCa-EA10 | 69 | White | 8.66 | 3+4 | 7 | III | None | Yes | | No | | |  | | |  |  |  |
| PCa-EA11 | 57 | White | 3.9 | 4+3 | 7 | II | -- | Yes | | No | | |  | | |  |  |  |
| PCa-EA12 | 65 | Asian | 6.46 | 4+5 | 9 | II | -- | Yes | | No | | |  | | |  |  |  |
| PCa-EA13 | 68 | White | 2.9 | 3+3 | 6 | -- | Fo | ? | | ? | | |  | | |  |  |  |
| PCa-EA14 | 65 | Hispanic | >10 | -- | -- | II | -- | Yes | | No | | |  | | |  |  |  |
| PCa-EA15 | 69 | White | 3.29 | 3+3 | 6 | -- | None | Yes | | No | | |  | | |  |  |  |
| PCa-EA16 | 56 | White | 6.4 | 4+3 | 7 | II | None | Yes | | No | | |  | | |  |  |  |
| PCa-EA17 | 55 | White | 6.82 | 3+4 | 7 | II | -- | Yes | | No | | |  | | |  |  |  |
| PCa-EA18 | 71 | White | 5.7 | 3+4 | 7 | II | None | Yes | | No | | |  | | |  |  |  |
| PCa-EA19 | 58 | White | 7.1 | 3+4 | 7 | -- | -- | ? | | ? | | |  | | |  |  |  |
| PCa-EA20 | 58 | White | 5.1 | 3+4 | 7 | II | None | Yes | | No | | |  | | |  |  |  |
| PCa-EA21 | 58 | White | 5 | 3+3 | 6 | -- | -- | ? | | ? | | |  | | |  |  |  |
| PCa-EA22 | 60 | White | 5.5 | 4+3 | 7 | III | -- | Yes | | -- | | |  | | |  |  |  |
| PCa-EA23 | 56 | White | N/A | 4+3 | 7 | -- | -- | | -- | | ? | | | ? | | |  | |
| PCa-EA24 | 66 | White | 6.2 | 3+4 | 7 | -- | None | Yes | | No | | | ?? | | |  |  |  |
| PCa-EA25 | 63 | White | N/A | -- | -- | III | None | No | | Yes | | | Hormonal | | |  |  |  |
| PCa-EA26 | 73 | White | 800 | -- | -- | IV | None | No | | Yes | | | Radiation, Hormonal, Chemo. | | |  |  |  |
| PCa-EA27 | 83 | White | 57 | 4+5 | 9 | IV | None | Yes | | Yes | | | Radiation, Hormonal, Chemo. | | |  |  |  |
| PCa-EA28 | 61 | White | N/A | 4+3 | 7 | IV | None | Yes | | -- | | | -- | | |  |  |  |
| PCa-EA29 | 69 | White | 43 | -- | -- | I | None | No | | Yes | | | Radiation, Hormonal, Chemo. | | |  |  |  |
| PCa-EA30 | 77 | White | N/A | -- | 6 | IV | None | Yes | | Yes | | | Radiation, Hormonal, Chemo. | | |  |  |  |
| PCa-EA31 | 61 | White | 4 | -- | 8 | IV | None | Yes | | Yes | | | Hormonal | | |  |  |  |
|  |  |  |  |  |  |  |  | |  | |  | | |  | | |  | |
| PCa-AA1 | 74 | Black | 5 | 4+3 | 7 | II | F3 | Yes | | No | | | None | | |  |  |  |
| PCa-AA2 | 53 | Black | 4 | 3+4 | 7 | II | None | Yes | | No | | | None | | |  |  |  |
| PCa-AA3 | 73 | Black | 710 | 2+4 | 6 | II | None | No | | No | | | Radiation | | |  |  |  |
| PCa-AA4 | 67 | Black | 23 | -- | -- | IV | None | No | | Yes | | | Radiation, Hormonal, Chemo. | | |  |  |  |
| PCa-AA5 | 59 | Black | N/A | 3+5 | 8 | -- | F1 | Yes | | -- | | | None | | |  |  |  |
| PCa-AA6 | 84 | Black | 13 | 4+4 | 8 | I | None | No | | No | | | Radiation Hormonal | | |  |  |  |
| PCa-AA7 | 71 | Black | 140 | -- | 7 | III | None | No | | No | | | Hormonal | | |  |  |  |
| PCa-AA8 | 57 | Black | N/A | -- | 8 | IV | None | No | | Yes | | | Hormonal | | |  |  |  |
| PCa-AA9 | 71 | 1/8 Native American | 7 | 3+4 | 7 | II | F0 | No | | No | | | Radiation, Hormonal | | |  |  |  |
| PCa-AA10 | 85 | Black | 630 | 4+4 | 8 | IV | None | Yes | | Yes | | | Radiation, Hormonal | | |  |  |  |
| PCa-AA11 | 79 | Black | 20 | 4+3 | 7 | I | None | No | | No | | | Radiation, Hormonal | | |  |  |  |
| PCa-AA12 | 77 | Black | 35 | 3+3 | 6 | -- | F10 | No | | No | | | Hormonal | | |  |  |  |
| PCa-AA13 | 73 | Black | 20 | 3+3 | 6 | -- | None | No | | No | | | Radiation | | |  |  |  |
| PCa-AA14 | 73 | Black | 12 | 3+3 | 6 | III | None | Yes | | Yes | | | Radiation, Hormonal | | |  |  |  |
| PCa-AA15 | 85 | Black | 8 | 4+3 | 7 | -- | None | No | | No | | | Hormonal | | |  |  |  |
| PCa-AA16 | 76 | Black | 29 | 3+3 | 6 | -- | F1 | No | | No | | | Radiation | | |  |  |  |
| PCa-AA17 | 62 | Black | 12 | -- | -- | -- | None |  | | ?? | | | ??? | | |  |  |  |
| PCa-AA18 | 66 | 1/4 Native American | 6 | 3+4 | 7 | III | None | Yes | | No | | | Radiation | | |  |  |  |
| PCa-AA19 | 84 | Black | 29 | 4+3 | 7 | -- | None | Yes | | Yes | | | Radiation, Hormonal | | |  |  |  |
| PCa-AA20 | 79 | Black | 24 | -- | -- | -- | None | -- | | -- | | | -- | | |  |  |  |
| PCa-AA21 | 59 | Black | 12 | -- | -- | -- | None | -- | | -- | | | -- | | |  |  |  |
| PCa-AA22 | 58 | 1/8 White; 1/8 Hispanic | 621 | -- | -- | -- | None | -- | | -- | | | -- | | |  |  |  |
| PCa-AA23 | 48 | Black | 5 | -- | -- | -- | None | -- | | -- | | | -- | | |  |  |  |
| PCa-AA24 | 77 | 1/4 Native American | 14 | -- | -- | -- | None | -- | | -- | | | -- | | |  |  |  |
| PCa-AA25 | 74 | Black | 5 | -- | -- | -- | None | -- | | -- | | | -- | | |  |  |  |
| PCa-AA26 | 62 | Black | 6 | -- | -- | -- | None | -- | | -- | | | -- | | |  |  |  |
| PCa-AA27 | 65 | Black | 17 | -- | -- | -- | None | -- | | -- | | | -- | | |  |  |  |
| PCa-AA28 | 80 | 1/8 White; 1/8 Native American | 19 | 3+2 | 5 | II | F02 | No | | No | | | Radiation, Hormonal | | |  |  |  |
| PCa-AA29 | 74 | 1/4 Native American | 3 | 4+3 | 7 | I | F1o | No | | No | | | Radiation, Hormonal | | |  |  |  |
| PCa-AA30 | 70 | Black | 6 | 3+4 | 7 | II | F0 | Yes | | Yes | | | Hormonal | | |  |  |  |
| PCa-AA31 | 80 | Black | 6 | 3+4 | 7 | II | None | Yes | | Yes | | | Hormonal | | |  |  |  |
| PCa-AA32 | 87 | Black | 83 | 4+4 | 8 | II | F0 | Yes | | Yes | | | Hormonal | | |  |  |  |
| PCa-AA33 | 48 | Black | 5.4 | 4+3 | 7 | II | ? | yes | | ? | | | ? | | |  |  |  |
| PCa-AA34 | 56 | Black | 58 | 5+4 | 9 | IV | ? | yes | | ? | | | ? | | |  |  |  |
| PCa-AA35 | 75 | Black | 4.5 | 3+3 | 6 | II | ? | yes | | ? | | | ? | | |  |  |  |
| PCa-AA36 | 67 | Black | 9.4 | 4+3 | 7 | II | ? | yes | | ? | | | ? | | |  |  |  |
| PCa-AA37 | 56 | Black | 7 | 4+3 | 7 | II | ? | yes | | ? | | | ? | | |  |  |  |
| PCa-AA38 | 51 | Black | 8.8 | 4+3 | 7 | III | ? | Yes | | ? | | | ? | | |  |  |  |
| PCa-AA39 | 54 | Black | 5.2 | 4+3 | 7 | II | ? | yes | | ? | | | ? | | |  |  |  |
| PCa-AA40 | 57 | Black | 9.3 | 4+3 | 7 | III | F1 | yes | | NA | | | No | | |  |  |  |
| PCa-AA41 | 62 | Black | 4.7 | 4+3 | 7 | III | F1 | yes | | NA | | | No | | |  |  |  |

African American patients are 100% black/AA unless otherwise noted. All sera from Bioserve are American-born (PCa-CC25 is from Poland, PCa-AA6 is from West Indies). PCa, prostate cancer, EA, European American; AA, African American.

“--“ indicates unknown information **F** = Family history of cancer

F1 – father, brother or grandfather with PCa, F2 – 2 members with PCa, F0 – member with other cancer, F02 – 2 members with other cancer (for example, sister, mother, father with lung cancer)
