# Supplementary material for: Racial differences in the expression of inhibitors of apoptosis (IAP) proteins in extracellular vesicles (EV) from prostate cancer patients
Source: PLoS One. 2017 Oct 5;12(10):e0183122. doi: 10.1371/journal.pone.0183122 (PMC5628787; doi:10.1371/journal.pone.0183122)
Supplement: S2 Table — (DOCX) [file pone.0183122.s006.docx]

**S2 Table. Rate of recurrence in both ethnicities and IAP expression in EV.**

|  | **Recurrence**  **Yes** | **Recurrence**  **No** | **Survivin** | **XIAP** | **cIAP-1/cIAP-2** |
| --- | --- | --- | --- | --- | --- |
| **PCa-CA** | **6 (27%)** | **16 (73%)** | **+++/++** | **++/++** | **+/++** |
| **PCa-AA** | **8 (36%)** | **14 (64%)** | **+++/++** | **+++/+++** | **++/++** |
